# Highly Selective Fluorescent Probe for the Detection of Copper (II) and Its Application in Live Cell Imaging

**DOI:** 10.1155/2019/8130767

**Published:** 2019-05-19

**Authors:** Zhihao Guo, Xiuji Wang, Pei Wei, Yihua Gao, Qin Li

**Affiliations:** Analysis Center, Guangdong Medical University, Dongguan 523808, China

## Abstract

The development of fluorescent methods for the detection of metal ions is of great importance due to their diverse environmental and biological roles. Herein, a rhodamine 6G-based off-on fluorescent probe (**L1**) with a *t*-butyl pyrrole moiety as the recognition site was designed and synthesized. Photophysical studies show that **L1** exhibits excellent sensitivity and selectivity towards Cu^2+^ to other metal ions in neutral acetonitrile aqueous media. Mechanism studies suggest that the recognition process may associate with a Cu^2+^ promoted hydrolysis reaction of **L1**. Furthermore, **L1** has been successfully applied in fluorescence imaging of Cu^2+^ ion in living cells.

## 1. Introduction

The exploration of detection methods for environmentally and biologically important metal ions is of great interest to researchers currently [1, 2]. Among these metal ions, Cu^2+^ receives great attention because copper is the third most abundant essential metal (after iron and zinc) in the human body and plays an important role in a variety of physiological processes. For example, copper is integrated into various proteins and metalloenzymes that perform basic metabolic functions [3]. Copper deficiency could produce osteoporosis, hyperthyroidism, and coronary heart disease [4]. However, excessive accumulation of copper can cause central nervous system damage and increase the risk of neurodegenerative diseases such as Alzheimer's, Parkinson's, Menken's, and Wilson's diseases [5–8]. Hence, development of sensitive and selective analysis methods for copper ion, especially those that could be utilized in bioimaging, is of great importance in the aspects of understanding the complex physiological functions of copper in the human body.

Traditionally, inductively coupled plasma atomic emission spectrometry (ICP-AES), atomic absorption spectroscopy (AAS), and inductively coupled plasma mass spectrometry (ICP-MS) are used to analyze copper ions [9–11]. However, these methods require sophisticated instruments and complicated and time-consuming processes of sample preparation. Thus, a simple and rapid detection method for quantifying copper ions is necessary. Fluorescence methods, with the advantages of sensitivity, simple operation, and real-time monitoring with fast response time, have been widely used in the detection of metal ions [12–21]. Due to the paramagnetic nature, Cu^2+^ was usually detected through fluorescence quenching of chemical sensors [22, 23], which may result in false-positive results and less-sensitive detection. Among the developed fluorophores, rhodamine derivatives, due to their excellent photophysical properties, such as large absorption coefficients, high fluorescence quantum yields, and long absorption and emission wavelengths, have attracted great attention from researchers [24–29]. In addition, it is well known that the fluorescence emission behaviors of rhodamine derivatives could be adjusted through a spirolactam ring-opening reaction. Spirocycle derivatives of rhodamine are colorless and nonfluorescent due to their nonconjugated structure. However, opening of the spirolactam ring, usually caused by metal ions, will produce intense fluorescence emission and a pink color change. Based on this spirolactam/ring-opened amide equilibrium of rhodamine, through introducing proper recognition ligands, researchers have developed many turn-on fluorescent sensors for metal ions [30–40].

Taking into account the criteria mentioned above, we herein incorporated a *t*-butyl pyrrole moiety into the rhodamine fluorophore to form a turn-on fluorescent probe **L1** for the detection of Cu^2+^. The molecular structure of **L1** was verified by ^1^H NMR, ^13^C NMR, and MS spectra. With the addition of Cu^2+^ to **L1** in a neutral CH_3_CN/H_2_O solution, metal-triggered ring-opening reaction of the spirolactam in **L1** took place, resulting in sensitive colorimetric response and fluorescence emission. Mechanism studies suggest that the detection process may associate with a Cu^2+^ promoted hydrolysis reaction of **L1**. Furthermore, fluorescence microscopy experiments demonstrated that **L1** could be used to image Cu^2+^ in living cells.

## 2. Materials and Methods

### 2.1. General Materials and Apparatus

All chemicals are purchased commercially and used directly without further purification. Deionized water was used throughout, and the pH was adjusted using diluted sodium hydroxide solution or hydrochloric acid. The pH value was measured with a Rex pHS-3E pH meter. Silica gel (200–300 mesh) was used for column chromatography. NMR spectra were obtained with a 600 MHz Bruker spectrometer, and tetramethylsilane (TMS) was used as the internal standard. High-resolution mass spectra were measured on an Agilent LCMS 6500 spectrometer. Absorbance spectra were measured on a Shimadzu UV-3101PC spectrometer. Measurements of fluorescence spectra were performed on a Shimadzu RF-5301PC spectrometer. Both excitation and emission slit widths were set at 3 nm. All experiments were operated at about 298 K.

### 2.2. Procedures of Sensing Experiments

A stock solution of probe **L1** (2 × 10^−4^ M) was prepared in CH_3_CN. Stock solutions of metal ions (2 × 10^−3^ M) were prepared in deionized water from their chloride salts or nitrate salt (Ag^+^). Working solution of **L1** (10 *μ*M, CH_3_CN/H_2_O, 1 : 1, v/v) was freshly prepared prior to spectroscopic experiments by diluting the high-concentration stock solution. In the sensing experiments, each time, a 3 mL working solution of **L1** (10 *μ*M, CH_3_CN/H_2_O, 1 : 1, v/v) was put in a quartz optical cell of 1 cm optical path length, and appropriate amounts of stock solutions of metal ions were added by a pipette. Spectral data were collected 2 min after the addition of the ions.

### 2.3. Synthesis of L1

To a 100 mL flask with three necks, rhodamine 6G hydrazine (2.8 mmol), which was synthesized according to reported methods [41], and 5-*tert*-butylpyrrole-2-carbaldehyde (3.3 mmol) were dissolved in 30 mL methanol. After addition of 0.1 mL acetic acid, the mixture was stirred and heated to reflux for 16 h. A pale yellow solid obtained was filtered off and washed using cold methanol. The solid was dried in vacuum and further purified by column chromatography (CH_2_Cl_2_/CH_3_OH = 150/1, v/v). Yield: 88.6%. ^1^H NMR (600 MHz, DMSO-*d*_6_), *δ* (ppm): 10.93 (s, 1H, CNHC), 8.16 (s, 1H, N=CH), 7.84 (d, *J* = 12 Hz, 1H, ArH), 7.50 (m, 2H, ArH), 6.94 (d, *J* = 12 Hz, 1H, ArH), 6.30 (s, 2H, ArH), 6.18 (s, 2H, ArH), 6.05 (d, *J* = 6 Hz, 1H, CCHCH), 5.77 (d, *J* = 6 Hz, 1H, CCHCH), 5.04 (t, *J* = 6 Hz, 2H, CH_3_CH_2_NH), 3.12 (q, *J* = 12 Hz, 4H, CH_3_CH_2_), 1.85 (s, 6H, ArCH_3_), 1.20 (m, 15H, CH_3_CH_2_, CCH_3_). ^13^C NMR (150 MHz, DMSO-*d*_6_), *δ* (ppm): 163.12, 151.96, 150.64, 147.66, 146.61, 141.63, 133.12, 128.41, 128.31, 126.64, 126.50, 123.24, 122.60, 118.11, 112.32, 105.03, 104.64, 95.91, 65.00, 37.44, 31.38, 29.98, 16.96, 14.13. HRMS: *m*/*z* calculated for M^+^, C_35_H_39_N_5_O_2_, 561.3104. Found: 562.3192 (M + 1).

### 2.4. Cell Culture and Fluorescence Imaging

The human breast adenocarcinoma cells MCF7 were cultured in DMEM supplemented with 10% fetal bovine serum at 37°C under an atmosphere containing 5% CO_2_. Before the experiments, the MCF7 cells were washed with PBS and then incubated with 10 *μ*M **L1** at 37°C for 30 min. After removal of excess probe and washed with PBS, the MCF7 cells were incubated with 20 *μ*M CuCl_2_ for another 30 min. The MCF7 cells were rinsed with PBS again and live-cell imaging was conducted using an EVOS FL Auto microscope.

## 3. Results and Discussion

**Scheme 1:**
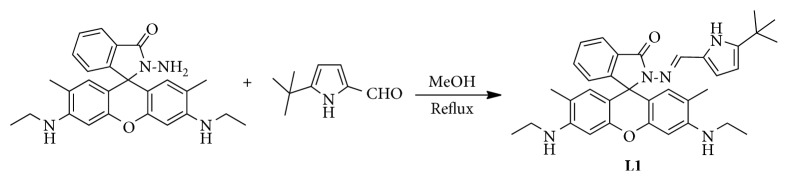
Synthesis of probe **L1**.

Compound **L1** was easily synthesized from rhodamine 6G through a two-step reaction (Scheme 1). After purification by column chromatography (CH_2_Cl_2_/CH_3_OH = 150/1, v/v), **L1** was obtained in an 88.6% yield. The molecular structure was verified by ^1^H NMR, ^13^C NMR, and MS spectra (Figures S1–S3). The *m*/*z* value of the molecular ion peak in the HRMS spectra was in good accordance with the accurate molecular weight with small derivation. Similar with other spirocycle derivatives of rhodamine, the solution of **L1** in neutral CH_3_CN/H_2_O media was colorless and weakly fluorescent, indicating that it existed mainly in the form of spirolactam. In addition, a characteristic spirocycle carbon chemical shift at 65.0 ppm in the ^13^C NMR spectra was observed, which further supported this estimation [42].

**Figure 1:**
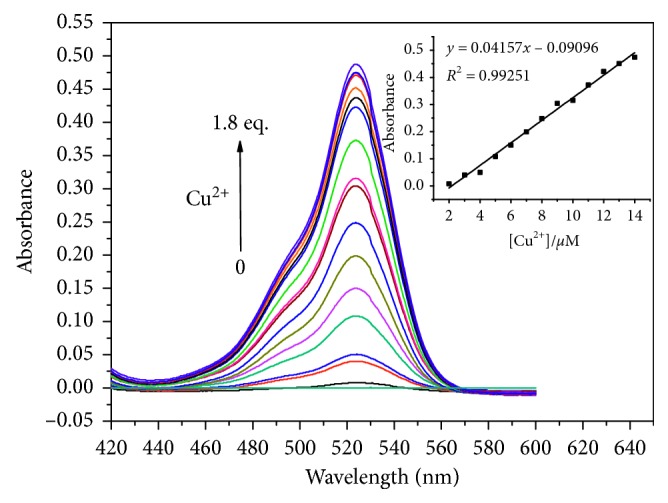
Absorbance spectra of **L1** (10 *μ*M) containing different concentrations of Cu^2+^ (0–18 *μ*M) in CH_3_CN/H_2_O solution (1 : 1, v/v). Inset: the absorbance at 525 nm as a function of Cu^2+^ concentration (2–14 *μ*M).

**Figure 2:**
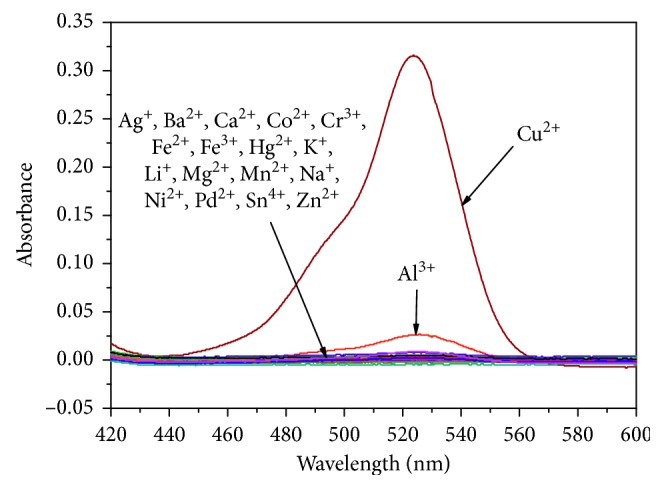
Absorbance spectra of **L1** (10 *μ*M) with addition of 10 *μ*M various metal ions in CH_3_CN/H_2_O solution (1 : 1, v/v).

The absorption spectrum of **L1** (10 *μ*M) in neutral CH_3_CN/H_2_O (1 : 1, v/v) solution is shown in Figure 1. No absorption peaks in the visible wavelength range was exhibited, suggesting that **L1** existed with the structure of spirolactam. Once the solution of Cu^2+^ was added, a new peak was detected at 525 nm. With the increase of Cu^2+^ concentration, the intensity of the peak was gradually enhanced, which could be interpreted as the transform from a spirolactam structure to a ring-opened form of **L1**. Correspondingly, the color of the solution changed from colorless to purple, which help to achieve the naked-eye recognition of Cu^2+^ ion. As shown in Figure 1, with the increasing concentration of Cu^2+^, the absorption value of **L1** (10 *μ*M) was found to increase linearly in the range of 2 *μ*M–14 *μ*M at 525 nm. The selective sensory studies of **L1** (10 *μ*M) in neutral CH_3_CN/H_2_O solution were then extended to other metal ions (Cr^3+^, Al^3+^, Fe^3+^, Ca^2+^, Ba^2+^, Co^2+^, Fe^2+^, Hg^2+^, Mg^2+^, Mn^2+^, Ni^2+^, Pd^2+^, Zn^2+^, Ag^+^, Li^+^, K^+^, Na^+^, and Sn^4+^). When 1 equiv metal ions were added into the relevant solution, only Cu^2+^ could induce a purple color and an obvious increase of absorbance at 525 nm (Figure 2). Al^3+^ exhibited weak absorption response, and the other metal ions showed almost no absorbance increase in the same condition. The results indicated that **L1** showed high selectivity towards Cu^2+^ in the detection of metal ions.

**Figure 3:**
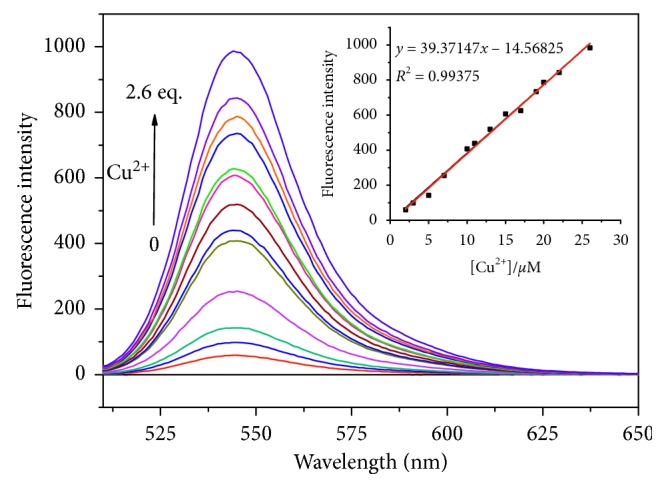
Fluorescence spectra of **L1** (10 *μ*M) containing different concentrations of Cu^2+^ (0–26 *μ*M) in CH_3_CN/H_2_O solution (1 : 1, v/v). Excitation was performed at 500 nm. Inset: fluorescence intensity at 545 nm as a function of Cu^2+^ concentration (2–26 *μ*M).

**Figure 4:**
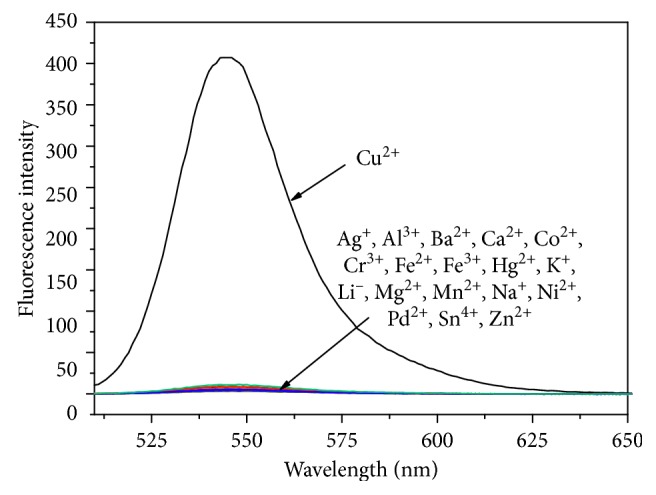
Fluorescence spectra of **L1** (10 *μ*M) with addition of 10 *μ*M various metal ions in CH_3_CN/H_2_O solution (1 : 1, v/v). Excitation was performed at 500 nm.

**Figure 5:**
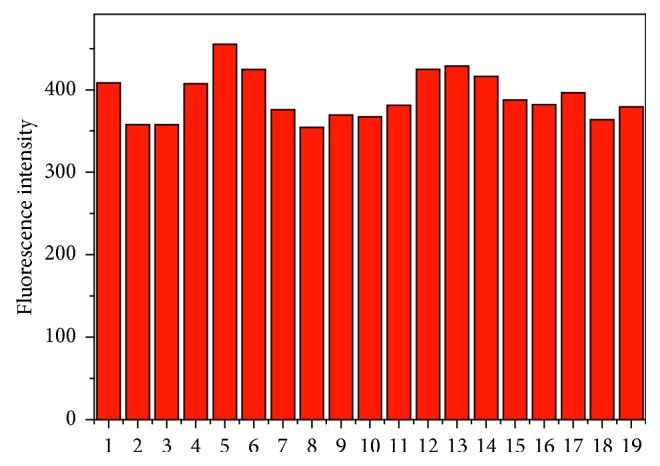
Fluorescence intensity at 545 nm of **L1** (10 *μ*M) with addition of 10 *μ*M Cu^2+^ in the absence (blank) and presence of various metal ions (50 *μ*M) in CH_3_CN/H_2_O solution (1 : 1, v/v). (1) blank, (2) Ag^+^, (3) Al^3+^, (4) Ba^2+^, (5) Ca^2+^, (6) Co^2+^, (7) Cr^3+^, (8) Fe^2+^, (9) Fe^3+^, (10) Hg^2+^, (11) K^+^, (12) Li^+^, (13) Mg^2+^, (14) Mn^2+^, (15) Na^+^, (16) Ni^2+^, (17) Pd^2+^, (18) Sn^4+^, and (19) Zn^2+^. Excitation was performed at 500 nm.

The fluorescence sensing behavior of **L1** for Cu^2+^ in neutral CH_3_CN/H_2_O solution (10 *μ*M, 1 : 1, v/v) was also investigated. When no Cu^2+^ was added, the free **L1** solution had little fluorescence at the excitation wavelength of 500 nm due to the spirocyclic form of its molecular structure. However, similar to the results of the absorption experiments, once the solution of Cu^2+^ was added, an obvious increase of the fluorescence intensity at 545 nm (Figure 3) could be detected, which could be ascribed to the generation of ring-opened conjugate structure in the rhodamine moiety. The fluorescence intensity at 545 nm of **L1** (10 *μ*M) was linear with the concentration of Cu^2+^ in the range of 2 *μ*M–26 *μ*M, and the detection limit of Cu^2+^ was calculated as 0.38 *μ*M (S/N = 3). The fluorescence quantum yield was calculated to be 0.19 in the presence of Cu^2+^ ion (26 *μ*M) by using rhodamine 6G as the standard (*Φ*_f_ = 0.94 in ethanol) [43]. The selectivity of **L1** towards Cu^2+^ over other cations was satisfactory. On excitation at 500 nm, no obvious spectral change of **L1** (10 *μ*M, CH_3_CN/H_2_O, 1 : 1, v/v) was observed with the addition of Cr^3+^, Al^3+^, Fe^3+^, Ca^2+^, Ba^2+^, Co^2+^, Fe^2+^, Hg^2+^, Mg^2+^, Mn^2+^, Ni^2+^, Pd^2+^, Zn^2+^, Ag^+^, Li^+^, K^+^, Na^+^, and Sn^4+^ (10 *μ*M). In contrast, more than 500-fold fluorescence intensity increase was detected when the same amount of Cu^2+^ ion was added (Figure 4). In order to further evaluate the selectivity of **L1** towards Cu^2+^ among other metal ions, the interference experiments were investigated. As shown in Figure 5, no obvious changes were observed in the Cu^2+^-induced fluorescence emission of **L1** when comparing the spectra data obtained in the presence and absence of other metal ions. These results indicated that **L1** could be used as a selective Cu^2^ fluorescent sensor without interference from other metal ions.

The influence of solution pH on the fluorescence response towards Cu^2+^ of **L1** was studied. As shown in Figure S4, no obvious fluorescence could be found for free **L1** between pH 4.0 and 8.0, indicating that the spirolactam structure still dominated in this pH range. However, with the addition of Cu^2+^, fluorescence change of **L1** was observed with different fluorescence enhancement efficiency under different pH values (Figure S5). A marked fluorescence response towards Cu^2+^ was achieved in a pH range from 7 to 8. These results indicated that **L1** could be used as a fluorescent probe for the detection of Cu^2+^ in physiological pH conditions.

The effects of reaction media for the detection of Cu^2+^ by **L1** were also studied. As shown in Figure S6, in the presence of 1 equiv. Cu^2+^, the fluorescence signal value of **L1** reached the maximum when acetonitrile content was at 50%–60%. As a result, 50% aqueous acetonitrile was employed in all optical experiments. The response time of the detection system on fluorescence emission was studied to evaluate the sensitivity of **L1** towards Cu^2+^. After addition of 1 equiv. Cu^2+^, the fluorescence intensity of **L1** increased rapidly and reached maximum after two minutes and did not increase with the prolongation of reaction time (Figure S7). These results indicated that **L1** was sensitive for the detection of Cu^2+^ in the aqueous acetonitrile solution, and 2 min was selected as the detection time in this research.

**Scheme 2:**
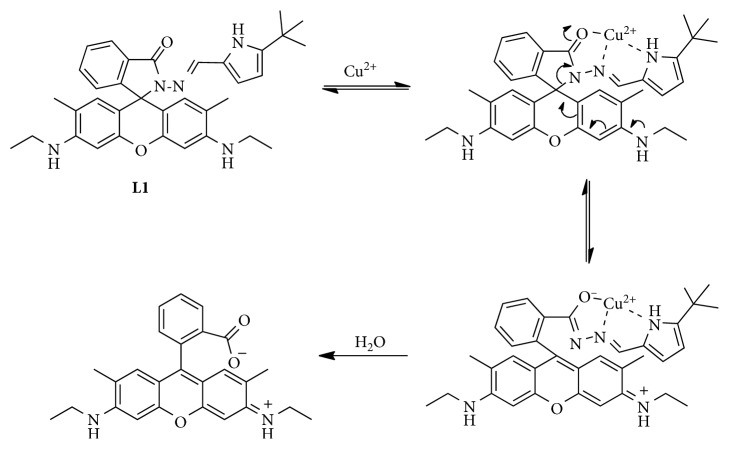
The proposed reaction mechanism of **L1** with Cu^2+^.

In order to illustrate the interaction mechanism between Cu^2+^ and **L1**, excess (10 equiv) Na_2_EDTA was added to the solution of **L1** in neutral CH_3_CN/H_2_O solution (10 *μ*M, 1 : 1, v/v) containing Cu^2+^ ion (10 *μ*M). No obvious decrease in the fluorescent intensity or color change was observed after the addition, indicating that the detection of Cu^2+^ ion by **L1** was an irreversible process (Figure S8). HRMS experiments were carried out to analyze the reaction products of Cu^2+^ and **L1**. The peak at *m*/*z* = 415.2025 was ascribed to rhodamine 6G (M + 1; *m*/*z* calculated for M^+^, C_26_H_26_N_2_O_3_, 414.1943), indicating rhodamine 6G as a final product (Figure S9). Moreover, the addition of Cu^2+^ into the solution of **L1** in pure CH_3_CN resulted in no fluorescent emission or color change. Based on these experimental results, a mechanism involving Cu^2+^-promoted redox hydrolysis of **L1** was proposed (Scheme 2), which was similar to that of the sensing towards Cu^2+^ by rhodamine B hydrazide [44].

**Figure 6:**
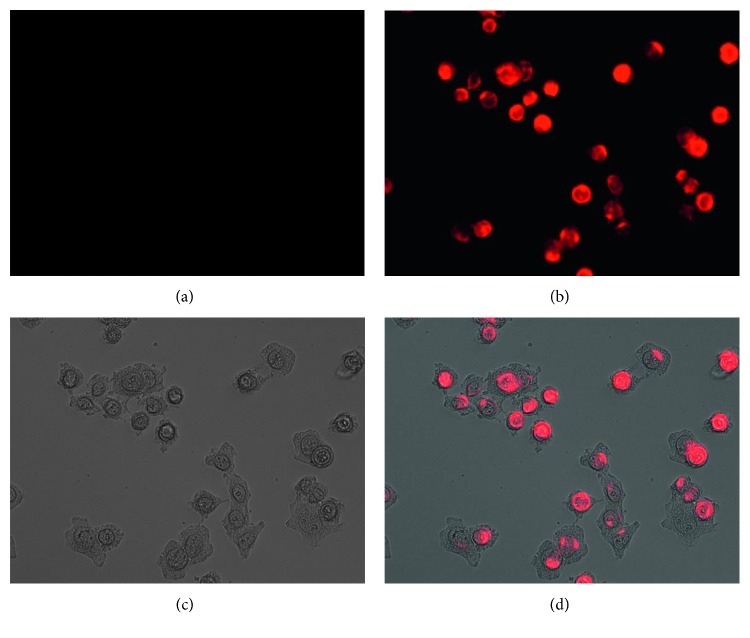
(a) Fluorescence image of MCF7 cells incubated with **L1** (10 *μ*M) for 30 min at 37°C. (b) Fluorescence image of MCF7 cells incubated with **L1** (10 *μ*M) for 30 min and then incubated with Cu^2+^ (20 *μ*M) for another 30 min at 37°C. (c) Brightfield image of cells shown in (b). (d) The overlay image of b and c.

For further studying the practical application of **L1** in the detection of Cu^2+^, fluorescence imaging experiments were carried out in MCF7 cells. The MCF7 cells were incubated with **L1** for 30 min and washed with PBS. As shown in Figure 6(a), no intracellular fluorescence was observed in the image. However, after subsequent treatment with CuCl_2_ at the same conditions for another 30 min, strong fluorescence in the MCF7 cells was observed (Figure 6(b)). These results suggest that **L1** could pass through the cell membrane and be used for the detection of Cu^2+^ in living cells. The cytotoxicity tests were studied by MTT assay with the concentrations of **L1** from 0 to 30 *μ*M (Figure S10). Experimental results showed that more than 95% of the MCF7 cells were viable after incubation with **L1** for 24 h at 37°C, indicating that **L1** has low cytotoxicity to cells in this dosage range.

## 4. Conclusion

In summary, a new colorimetric and fluorescent probe **L1** for Cu^2+^ was developed through the combination of rhodamine 6G and a pyrrole moiety. As a turn-on fluorescent probe, **L1** exhibited excellent sensitivity and selectivity for Cu^2+^ detection in acetonitrile aqueous media with a low detection limit. Moreover, fluorescence bioimaging experiments confirmed that **L1** could be used to detect intracellular Cu^2+^ in living cells.

## Data Availability

The data used to support the findings of this study are available from the corresponding author upon request.

## Supplementary Materials

Supplementary Materials Figure S1: ^1^H NMR of compound **L1** (600 MHz, DMSO-*d*_6_). Figure S2: ^13^C NMR of compound **L1** (150 MHz, DMSO-*d*_6_). Figure S3: high-resolution mass spectra of **L1**. Figure S4: effect of pH on fluorescence intensity at 545 nm of **L1** (10 *μ*M) in CH_3_CN/H_2_O solution (1 : 1, v/v). Excitation was performed at 500 nm. Figure S5: effect of pH on fluorescence intensity at 545 nm of **L1** (10 *μ*M) in the presence of 10 *μ*M Cu^2+^ in CH_3_CN/H_2_O solution (1 : 1, v/v). Excitation was performed at 500 nm. Figure S6: effect of acetonitrile content on fluorescence intensity of **L1** (10 *μ*M) in the presence of 10 *μ*M Cu^2+^ in CH_3_CN/H_2_O solution. Excitation was performed at 500 nm. Figure S7: time course of the response of **L1** (10 *μ*M) to 1 equiv. of Cu^2+^ in CH_3_CN/H_2_O solution. Figure S8: fluorescence spectra of **L1** (10 *μ*M) with addition of 10 *μ*M Cu^2+^ in the absence and presence of 10 equiv. Na_2_EDTA in CH_3_CN/H_2_O solution (1 : 1, v/v). Figure S9: mass spectrum for the reaction products of Cu^2+^ and **L1** in CH_3_CN/H_2_O solution (1 : 1, v/v). Figure S10: the viability of MCF7 cells incubated with **L1** of different concentrations.
