# Supplementary material for: Highly Selective Fluorescent Probe for the Detection of Copper (II) and Its Application in Live Cell Imaging
Source: J Anal Methods Chem. 2019 May 19;2019:8130767. doi: 10.1155/2019/8130767 (PMC6545799; doi:10.1155/2019/8130767)
Supplement: Supplementary Materials — Figure S1: 1H NMR of compound L1 (600 MHz, DMSO-d 6). Figure S2: 13C NMR of compound L1 (150 MHz, DMSO-d 6). Figure S3: high-resolution mass spectra of L1. Figure S4: effect of pH on fluorescence intensity at 545 nm of L1 (10 μM) in CH3CN/H2O solution (1 : 1, v/v). Excitation was performed at 500 nm. Figure S5: effect of pH on fluorescence intensity at 545 nm of L1 (10 μM) in the presence of 10 μM Cu2+ in CH3CN/H2O solution (1 : 1, v/v). Excitation was performed at 500 nm. Figure S6: effect of acetonitrile content on fluorescence intensity of L1 (10 μM) in the presence of 10 μM Cu2+ in CH3CN/H2O solution. Excitation was performed at 500 nm. Figure S7: time course of the response of L1 (10 μM) to 1 equiv. of Cu2+ in CH3CN/H2O solution. Figure S8: fluorescence spectra of L1 (10 μM) with addition of 10 μM Cu2+ in the absence and presence of 10 equiv. Na2EDTA in CH3CN/H2O solution (1 : 1, v/v). Figure S9: mass spectrum for the reaction products of Cu2+ and L1 in CH3CN/H2O solution (1 : 1, v/v). Figure S10: the viability of MCF7 cells incubated with L1 of different concentrations. [file 8130767.f1.pdf]

Supporting Information

Highly Selective Fluorescent Probe for the Detection of Copper (II) and Its Application in Live Cell Imaging

Zhihao Guo#, Xiuji Wang#, Pei Wei, Yihua Gao*, Qin Li
Analysis Center, Guangdong Medical University, Dongguan, 523808, P. R. China
#These authors contributed equally to this work
*Corresponding author, Tel.: +86 76922896078; E-mail: gaoyh@gdmu.edu.cn

1. Structure characterization

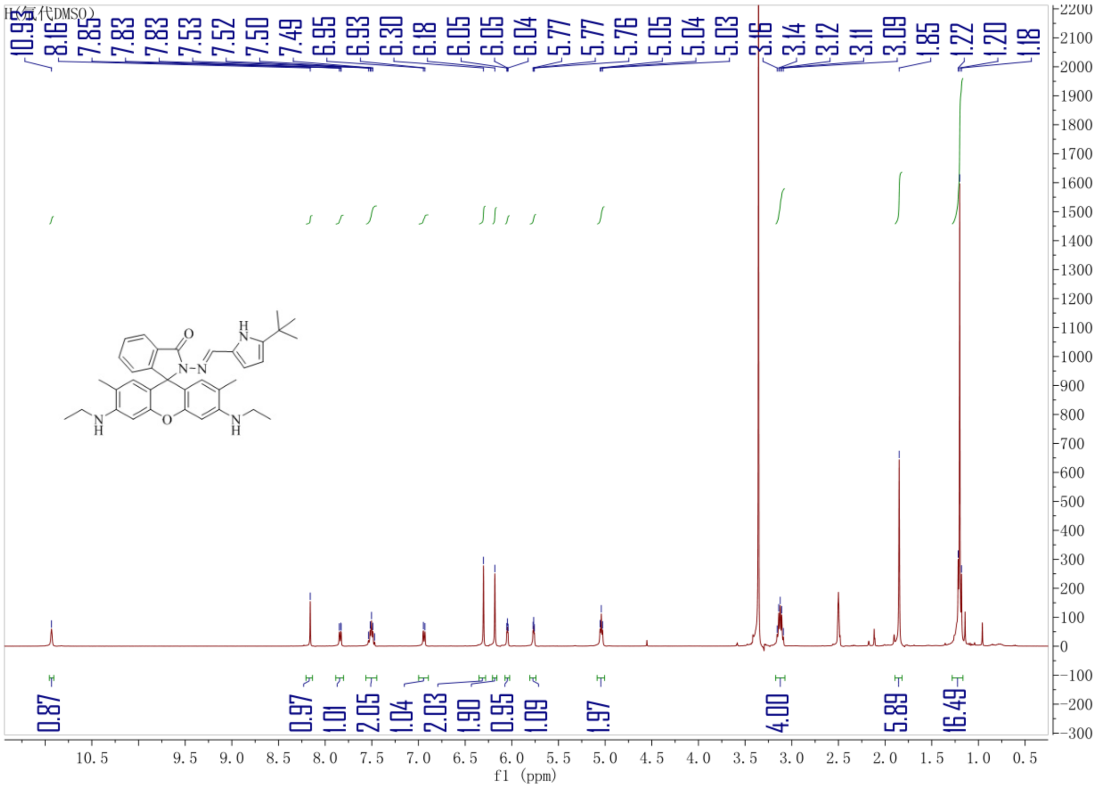

Figure S1 ^{1}H NMR of compound **L1** (600 MHz, DMSO-d$_6$).

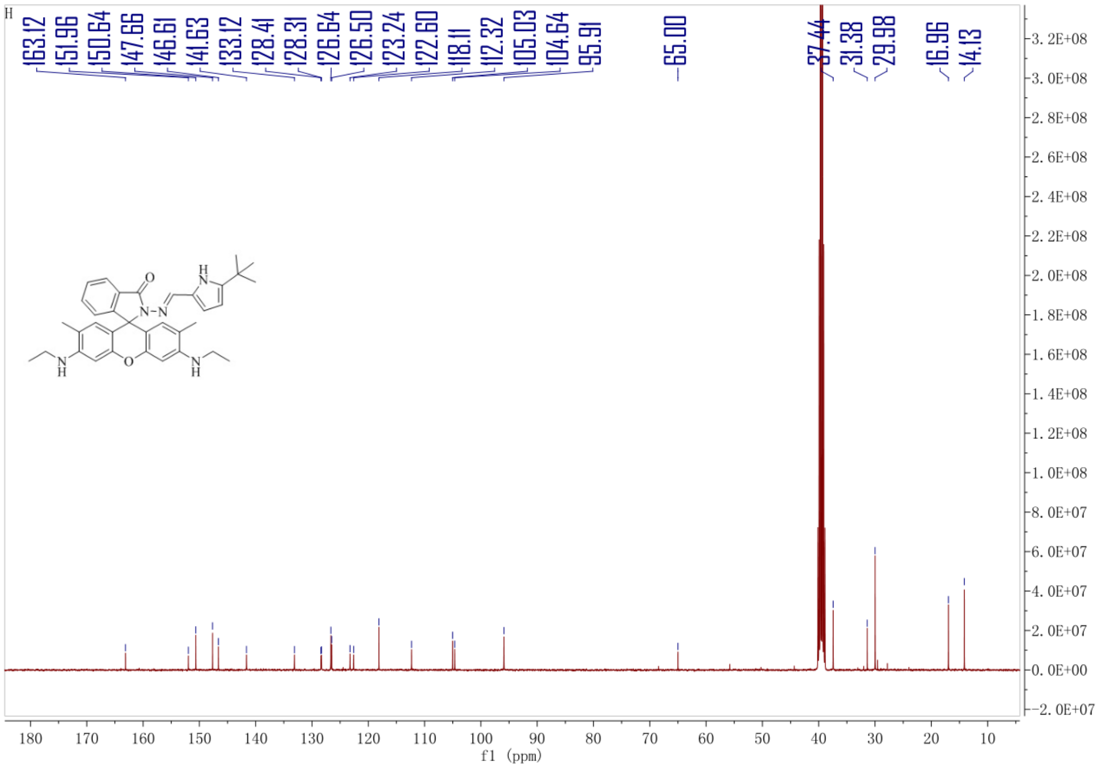

Figure S2 ^{13}C NMR of compound **L1** (150 MHz, DMSO-d$_6$).

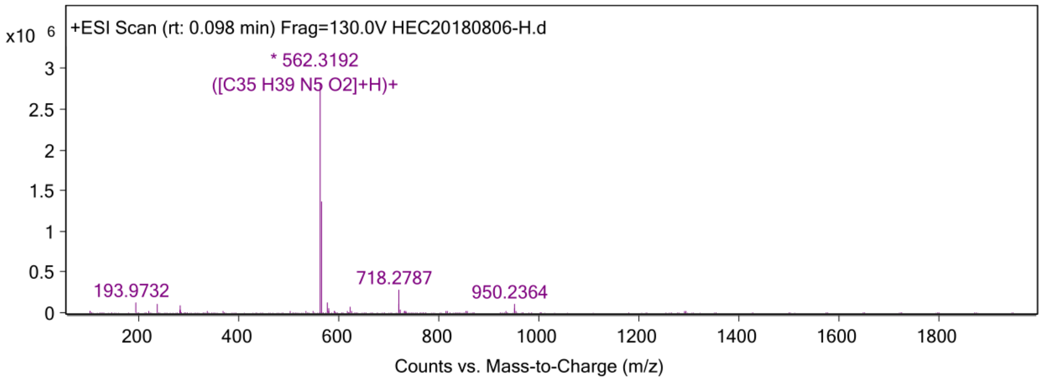

Figure S3 High resolution mass spectra of **L1**.

2. Additional spectrum

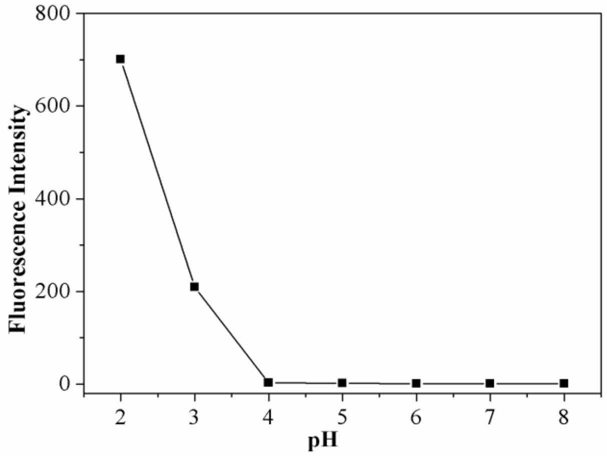

Figure S4 Effect of pH on fluorescence intensity at 545 nm of **L1** (10 μM) in CH_3CN/H_2O solution (1:1, v/v). Excitation was performed at 500 nm.

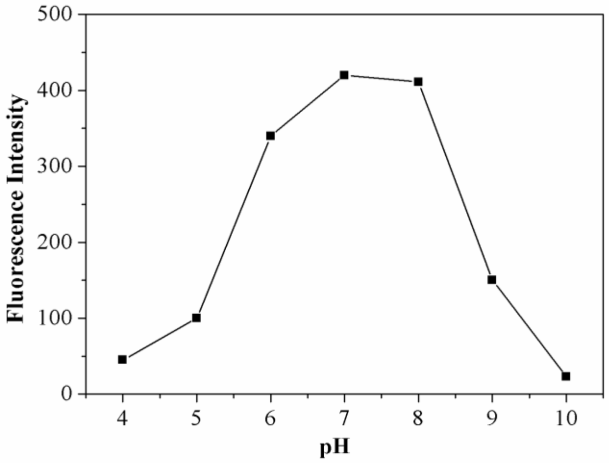

Figure S5 Effect of pH on fluorescence intensity at 545 nm of **L1** (10 μM) in the presence of 10 μM Cu^{2+} in CH_3CN/H_2O solution (1:1, v/v). Excitation was performed at 500 nm.

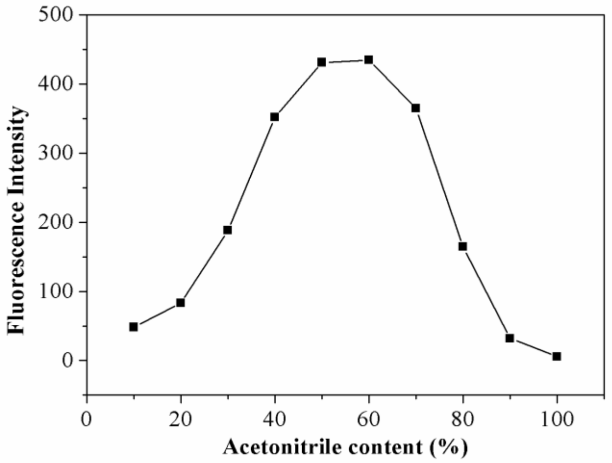

Figure S6 Effect of acetonitrile content on fluorescence intensity of **L1** (10 μM) in the presence of 10 μM Cu^{2+} in CH_3CN/H_2O solution. Excitation was performed at 500 nm.

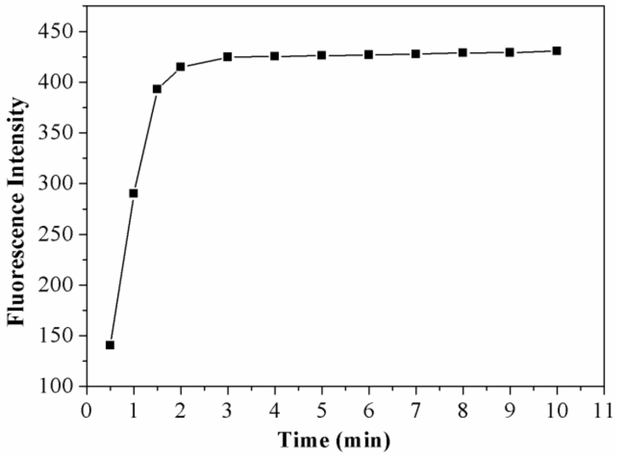

Figure S7 Time course of the response of **L1** (10 μM) to 1 equiv. of Cu^{2+} in CH_3CN/H_2O solution.

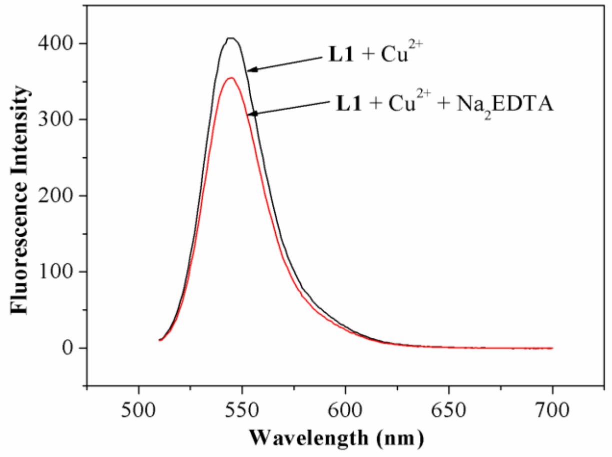

Figure S8 Fluorescence spectra of **L1** (10 μM) with addition of 10 μM Cu^{2+} in the absence and presence of 10 equiv. Na_2EDTA in CH_3CN/H_2O solution (1:1, v/v).

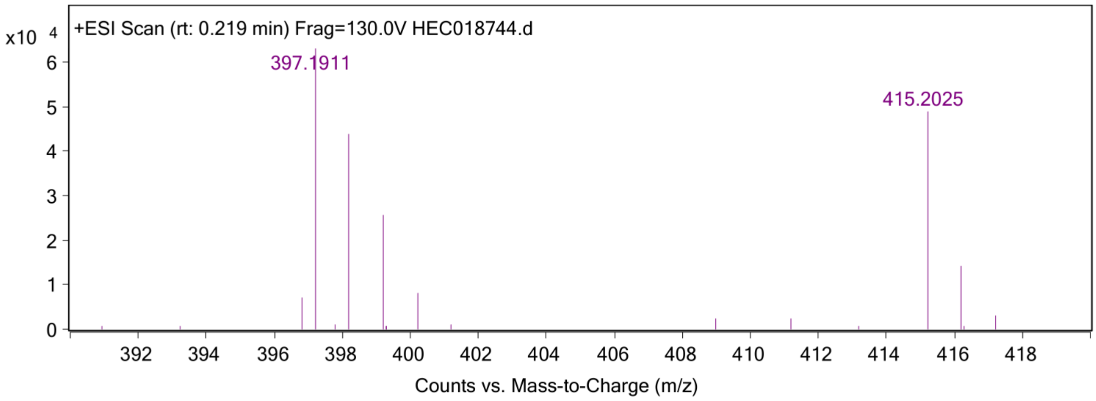

Figure S9 Mass spectrum for the reaction products of Cu^{2+} and **L1** in CH_3CN/H_2O solution (1:1, v/v).

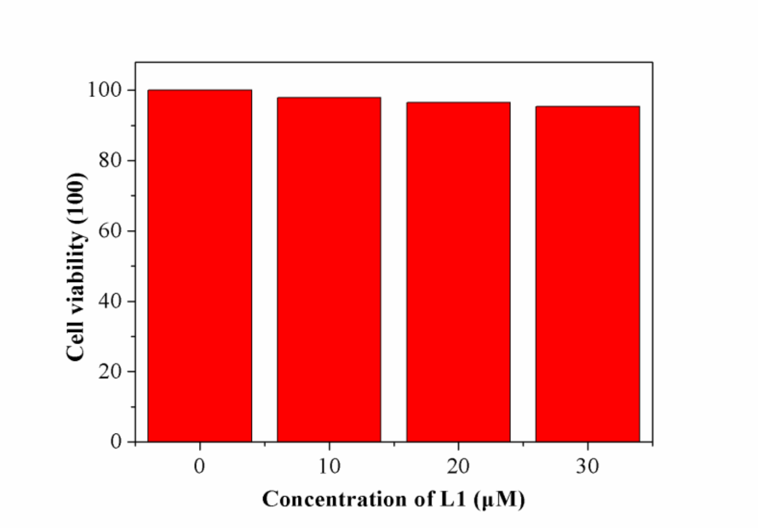

Figure S10 The viability of MCF7 cells incubated with **L1** of different concentrations.
